# Primary Cardiac Synovial Sarcoma (PCSS): Clinicopathologic Features of 6 Cases and Literature Comparison

**DOI:** 10.1155/crp/5272035

**Published:** 2026-04-13

**Authors:** Yi-Xiang Cai, Sheng-Jun Liu, Qi Sun, Hui Li, Xiao-Le Meng

**Affiliations:** ^1^ Department of Pathology, Zhong Shan Hospital (Xiamen Branch), Fudan University, Xiamen, 361015, China, fudan.edu.cn; ^2^ Department of Pathology, Xiang’an Hospital of Xiamen University, School of Medicine, Xiamen University, Xiamen, 361102, China, xmu.edu.cn; ^3^ Department of Pathology, Zhong Shan Hospital, Fudan University, Shanghai, 200032, China, fudan.edu.cn; ^4^ Clinical Research Center for Precision Medicine of Abdominal Tumor of Fujian Province, Xiamen, 361015, China

**Keywords:** clinicopathologic features, primary cardiac synovial sarcoma (PCSS), SS18 (SYT), TLE1

## Abstract

Primary cardiac synovial sarcoma (PCSS) is an exceedingly rare tumor. This study presents a comprehensive analysis of six novel PCSS cases identified within our institutional cohort, compared with published literature cohorts, focusing on their clinical presentations, histopathological features, immunohistochemical and molecular characteristics, therapeutic interventions, and prognosis. Our institutional cohort included five male and one female patients, with a median age of 44 years. Presenting symptoms included dyspnea, chest tightness, back pain, and syncope. Tumors were located in the pericardium (four cases) and the cardiac wall (two cases). The tumor size ranged from 2.0 to 14.5 cm. Histopathologically, four cases were monophasic and two were biphasic. Immunohistochemical analysis revealed consistent expression of TLE1, vimentin, and BCL‐2. Molecular analysis confirmed the presence of the *SS18–SSX* gene fusion through fluorescence in situ hybridization (FISH) in five cases, whereas one FISH‐negative case was positive for the *SS18–SSX1* gene fusion through next‐generation sequencing (NGS). All patients underwent surgical intervention (tumor excision) followed by adjuvant chemotherapy with doxorubicin and ifosfamide. At follow‐up, four patients were alive without disease and two had died. This case series highlights the clinicopathologic and molecular features of PCSS. Overall, *SS18 (SYT)* gene rearrangement and TLE1 expression are crucial diagnostic markers for differentiating PCSS from other neoplasms.

## 1. Introduction

Primary cardiac synovial sarcoma (PCSS) is a rare malignant soft tissue tumor that accounts for less than 5% of all primary cardiac sarcomas [1, 2]. Historically, these tumors are designated “synovial sarcomas” due to their histopathological resemblance to synovium. However, synovial sarcoma lacks a histogenetic relationship with synovial tissue; rather, its origin is more plausibly attributed to primitive mesenchymal tissue, which frequently exhibits varying degrees of epithelioid differentiation [3].

The diagnosis and treatment of PCSS remain significant challenges due to its rarity and nonspecific clinical manifestations [4]. PCSS patients typically present with nonspecific cardiopulmonary symptoms, including shortness of breath, dyspnea, chest pain, and cough [5]. These symptoms overlap substantially with those of more prevalent cardiovascular diseases [2], complicating early diagnosis. Furthermore, the histopathological features of PCSS closely mimic those of other cardiac neoplasms, contributing to diagnostic delays and compromised treatment.

Diagnosing synovial sarcoma relying exclusively on hematoxylin and eosin (H&E) staining and immunohistochemistry (IHC) remains a formidable diagnostic challenge. Recent advancements in molecular biology have markedly enhanced diagnostic accuracy. Synovial sarcoma is characterized by the chromosomal translocation t (X; 18) (*SS18–SSX*) in over 95% of cases [6]. This translocation results in the expression of the *SS18–SSX* oncogenic fusion protein, thereby driving the occurrence and development of the sarcoma [7]. The primary fusion variants of synovial sarcoma are *SS18–SSX1 (SYT–SSX1)* and *SS18–SSX2 (SYT–SSX2)*, whereas *SS18–SSX4 (SYT–SSX4)* is relatively less common [8]. Consequently, the detection of *SS18* gene rearrangement via fluorescence in situ hybridization (FISH), real‐time polymerase chain reaction (RT‐PCR), or next‐generation sequencing (NGS) has been established as the diagnostic gold standard [5] for synovial sarcoma.

This study presents six novel cases of PCSS and a comparison of 112 PCSS cases from prior literature. We systematically evaluated the clinical, histopathological, immunohistochemical, and molecular characteristics, along with therapeutic interventions and prognostic outcomes, providing a comprehensive analysis from clinical manifestations to genetic profiles. A graphical abstract summarizing key content is provided in Supporting Figure 1.

## 2. Materials and Methods

### 2.1. Case Selection and Clinical Data

First, we retrospectively reviewed the medical records at our hospital spanning from January 1, 2020, to March 31, 2025. We searched the local database for cases with a documented diagnosis of cardiac synovial sarcoma. Six cases were identified: four originated from the pericardium and two from the cardiac wall. We excluded tumors originating from outside the heart. Clinical data were extracted from the reviewed medical records, and diagnoses were established through comprehensive pathological examinations. Immunohistochemical staining and molecular analyses were performed for disease differential diagnosis. Patients were subjected to continuous follow‐up. The validity of the diagnosis was discussed in each case. This part was conducted in accordance with the Declaration of Helsinki and received approval from the appropriate ethical committees.

In the second part of the study, we searched the PubMed database using the keyword “cardiac synovial sarcoma.” Studies were included if they met the following inclusion criteria: (1) pathologically confirmed diagnosis of PCSS and (2) tumor origin within the fibrous skeleton of the heart, the cardiac wall, or the pericardium. Studies were excluded if they met any of the following exclusion criteria: (1) cardiac metastasis of extracardiac synovial sarcoma; (2) cases without a definitive diagnosis of synovial sarcoma; (3) duplicate case reports; or (4) unavailability of full‐text articles. To mitigate the impact of full‐text unavailability, patient data extracted from published review articles were included where applicable.

**FIGURE 1:**
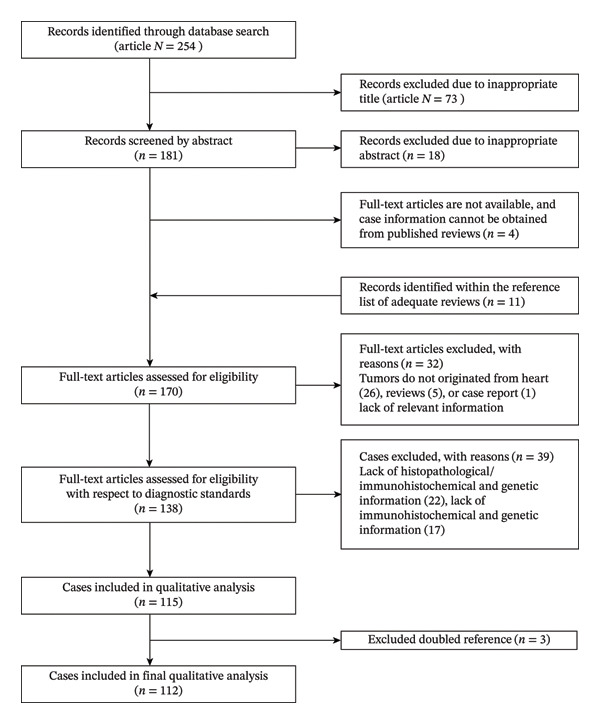
Flow diagram of literature comparison.

The search was conducted on January 20, 2025, and retrieved 254 hits (Figure 1). For 73 hits, the title and for 18 the abstract clearly indicated that the manuscript did not focus on the topic of this review. A further four articles were excluded due to a lack of relevant or original information. On top of these 159 articles, we further checked the reference list and found 11 additional manuscripts that align with our topic. Of the remaining 170 papers, a further 32 were excluded by screening full‐text articles. A total of 154 cases identified from 138 published articles were then evaluated concerning diagnostic standards. *SS18* gene rearrangement represents the diagnostic gold standard for synovial sarcoma. For cases lacking molecular genetic data, immunohistochemical findings may serve as alternative diagnostic criteria. Cases were excluded only when both molecular genetics and IHC information were unavailable. Of 154 initially eligible cases, 39 were excluded due to a lack of relevant or original data. Hence, 112 cases were included in the final literature comparison. Two researchers independently screened titles/abstracts and extracted data from full texts. Discrepancies were resolved through consensus or adjudication by a third senior researcher. All 112 cases included were arranged by publication time frame in Supporting Table 1.

### 2.2. Immunohistochemistry (IHC)

Specimens were fixed in 10% neutral buffered formalin, subsequently processed via a graded ethanol dehydration series, and embedded in paraffin. Four‐micrometer‐thick sections were prepared and stained with H&E for histological evaluation. IHC using diaminobenzidine (DAB) chromogen was performed with the EnVision two‐step detection system. Positive and negative controls were incorporated into each staining procedure to validate results. Primary antibodies sourced from Dako, Fuzhou Maxim Biotech, Leica Biosystems, and Shanghai Long Island Biotech were utilized.

### 2.3. FISH

FISH analysis was performed using the Vysis *SS18* Dual Color, Break Apart Rearrangement Probe (Abbott), comprising a ∼650‐kb spectrum red–labeled probe distal to the *SS18* gene and a 1044‐kb spectrum green–labeled probe proximal to the *SS18* gene; a minimum of 100 tumor cells per sample were analyzed, with a positive result defined by the observation of distinct red and green signal separation, indicative of an *SS18* gene rearrangement.

### 2.4. NGS

NGS was employed to detect *SS18–SSX* fusion transcripts, employing a targeted RNA sequencing panel for solid tumor characterization. The mutations and chromosomal rearrangements in genes involved in tumors were tested using NGS on the Ion Torrent Personal Genome Machine (Life Technologies) with the ThyroSeq v2 assay.

### 2.5. Statistical Analysis

The patient data included case sex, age, symptoms, medical history, location, tumor size, histopathology, immunohistochemical analysis, treatment, prognosis, and survival time. The histopathology data were divided into three categories for statistical analysis: monophasic, biphasic, and undifferentiated. Data collection was performed using Microsoft Excel, and statistical analyses were executed with IBM SPSS Statistics.

## 3. Results

### 3.1. Original Case Series

**FIGURE 2:**
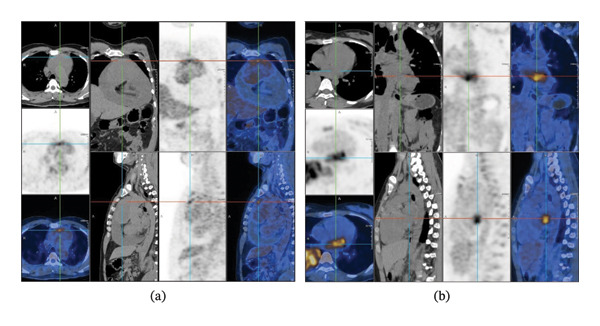
PET–CT scan of primary cardiac synovial sarcoma patients. (a) (Case 1) PET–CT scan demonstrated a heterogeneous enhancing soft tissue mass within the pericardial cavity. (b) (Case 2) PET–CT scan demonstrated a hypodense lesion with increased abnormal glucose metabolism in the left atrium and left ventricle.

Six novel cases were diagnosed within our institution between January 1, 2020, and August 31, 2024, consisting of five male and one female patients. The clinicopathologic findings are detailed in Table 1. The age distribution ranged from 21 to 53 years, with a median age of 44 years. The symptoms included dyspnea (*n* = 4), chest tightness (*n* = 3), back pain (*n* = 3), and syncope (*n* = 1). No lower extremity edema was found on examination, but imaging showed asymmetric pericardial effusion in five patients. An elevation in NT‐proBNP (117–1941 pg/mL; normal 0–100 pg/mL) and cardiac troponin T (cTnT) levels (0.005–0.060 ng/mL; normal 0–0.014 ng/mL) were observed in a majority of cases. Positron emission tomography–computed tomography (PET–CT) scans were conducted for all patients. The maximum standardized uptake values (SUV_max_) ranged from 2.7 to 10.3. Pericardial involvement was identified in Cases 1, 3, 4, and 5. Case 2 presented with left atrial and ventricular infiltration, whereas Case 6 exhibited right atrial and ventricular tumor extension. Case 1 exhibited a pericardial mass with focally increased abnormal glucose metabolism, showing local invasion at the superior vena cava junction. The mean CT value was 47.4 Hounsfield units (HUs), and the SUV_max_ value was 5.3 (Figure 2(a)). Case 2 presented a hypodense lesion originating from the left atrium and extending into the left ventricle. The mean CT value was 29.9 HU, and the SUV_max_ value was 6.8 (Figure 2(b)).

**TABLE 1:** Clinical and pathological features of six new cases.

*N*	Sex/age	Location	Size (cm)	Symptoms	Subtype	Genetics
1	M/49	Pericardium	10.96	Chest tightness, dyspnea	Monophasic	*SS18* (FISH)
2	F/21	Left atrium	5.19	Back pain, dyspnea	Biphasic	*SS18* (NGS)
3	M/36	Pericardium	4.65	Syncope episode, unconsciousness	Monophasic	*SS18* (FISH)
4	M/51	Pericardium	3.29	Chest tightness, dyspnea	Monophasic	*SS18* (FISH)
5	M/51	Pericardium	12.00	Chest tightness, dyspnea	Monophasic	*SS18* (FISH)
6	M/53	Right ventricle	6.51	Persistent back pain	Biphasic	*SS18* (FISH)

**FIGURE 3:**
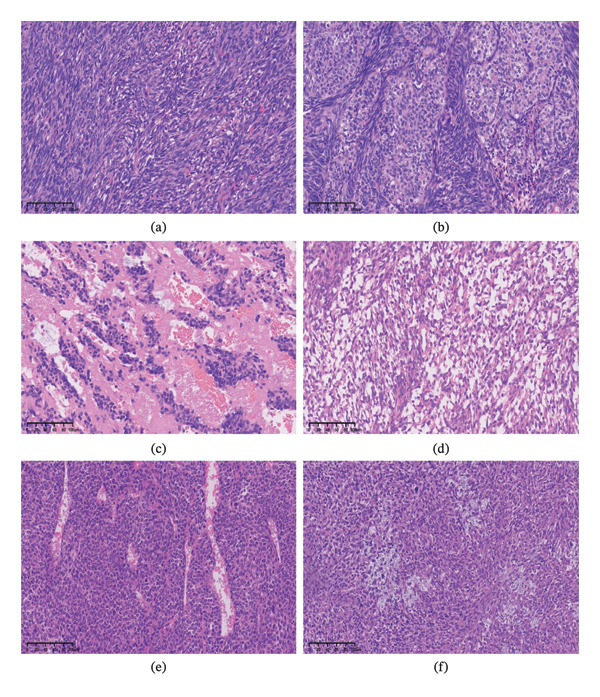
Histopathological characteristics of primary cardiac synovial sarcoma. (a) Monophasic cardiac synovial sarcoma, with interwoven, fascicular, or herringbone patterns. (b) Biphasic cardiac synovial sarcoma. The tumor consisted of a mixture of spindle cell and epithelial components. (c) Abundant sinusoids in the mesenchyme. (d) Sparse cell density in monophasic cardiac synovial sarcoma, with interstitial edema, mucoid degeneration, eosinophilic fibroblast, and scattered inflammatory cell. (e) Monophasic cardiac synovial sarcoma presented hemangiopericytoma‐like structure. (f) Tumor cells exhibited focal myxoid degeneration. The scale bars indicate 100 µm.

Grossly, the tumors presented as well‐defined, large, ashen, or red–gray masses, enclosed by a fibrous pseudocapsule. The cut surfaces were grayish white and soft. The maximum tumor diameter ranged from 2.0 cm to 14.5 cm. Four cases (Cases 1, 3, 4, and 5) were classified as monophasic cardiac synovial sarcoma, which is primarily composed of monomorphic spindle cells. Tumor cells were densely arranged, forming interwoven, fascicular, or herringbone patterns (Figure 3(a)). Two patients (Cases 2 and 6) were classified as biphasic cardiac synovial sarcoma, characterized by varying proportions of spindle cells and epithelial cells (Figure 3(b)). In the monophasic cases, the stroma consistently showed thin‐walled vascular proliferation and abundant sinusoids (Figure 3(c)). Furthermore, histologic overlap with inflammatory myofibroblastic tumor (IMT) was observed in some regions, characterized by interstitial edema, mucoid degeneration, eosinophilic fibroblasts, and scattered inflammatory cells (Figure 3(d)). Besides, one case displayed a hemangiopericytoma‐like vascular pattern (Figure 3(e)). Interestingly, focal mucoid degeneration was identified in discrete perivascular and intratumoral compartments, suggesting potential stromal–epithelial interactions (Figure 3(f)). In addition, a highly variable mitotic rate was observed in both monophasic and biphasic subtypes, directly correlating with an elevated Ki‐67 index, reflecting the differential proliferative potential of tumor cells and their aggressive biological behaviors.

**FIGURE 4:**
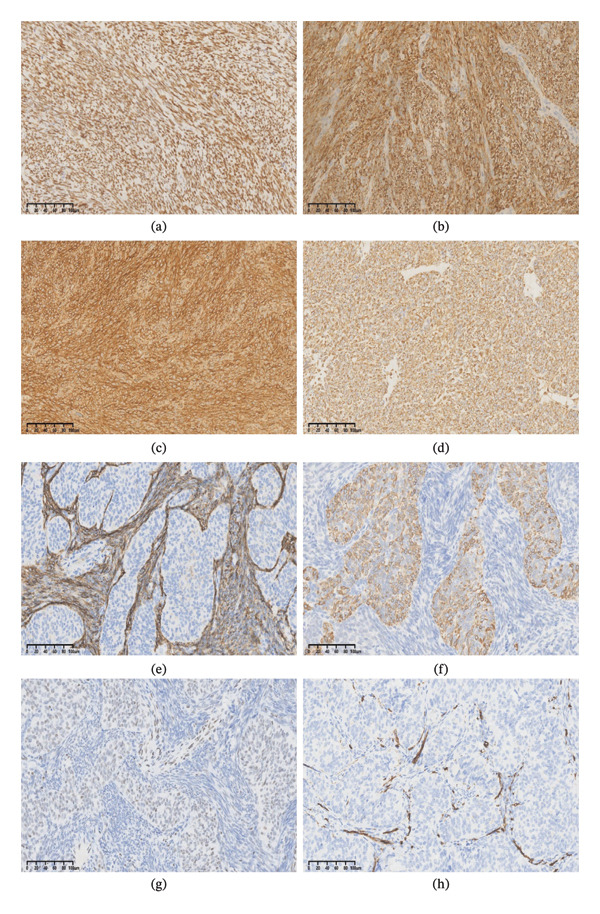
Immunohistochemical features of primary cardiac synovial sarcoma. (a) TLE1 exhibited intense positive nuclear staining in all our cases. (b) The tumor cells were positive for BCL‐2. (c) Both epithelial and spindle tumor cells were positive for CD99. (d) Vimentin positivity was observed in almost all tumor cells. (e) The spindle cells were immune‐positive for CD56 but were negative in epithelial components. (f) The epithelial components exhibited positive staining for CK but were negative in spindle cells. (g) INI‐1 (SMARCB1) immunoreactivity was detected in the epithelial components but was negative in spindle cells. (h) CD34 was positive in stroma blood vessels but was negative in tumor cells. The scale bars indicate 100 µm.

Immunohistochemically, our cases showed strong nuclear expression of TLE1 (*n* = 6/6; Figure 4(a)). BCL‐2 exhibited strong cytoplasmic expression in spindle cells (*n* = 3/3; Figure 4(b)). CD99 showed uniform positivity in both spindle and epithelial tumor components (*n* = 3/3; Figure 4(c)), further supporting this diagnosis. Vimentin positivity, which is indicative of mesenchymal origin, was diffusely observed in tumor cells (6/6 cases; Figure 4(d)). Subtype‐specific immunophenotypic patterns were clearly evident in the tumor samples. Biphasic tumors (Cases 2 and 6) displayed strong cytokeratin (CK) expression in epithelial components and CD56 positivity in spindle cell components (Case 2; Figure 4(e), 4(f)). Monophasic tumors (Cases 1 and 3–5) exhibited focal or absent CK/epithelial membrane antigen (EMA) expression in spindle cell regions. INI‐1 (SMARCB1) immunoreactivity was retained in epithelial components (Figure 4(g)), excluding diagnoses associated with INI‐1 loss, such as epithelioid sarcoma [9]. CD34 highlighted tumor‐associated vasculature but was negative in tumor cells (*n* = 6/6; Figure 4(h)). All cases were negative for myogenin, MyoD1, SMA, desmin, S100, and STAT6, ruling out myogenous [10], neural [11], and solitary fibrous [12] tumors. The Ki‐67 ranged from 10% to 70%, with a mean value of 48.3%. WT‐1 was negative in all cases, excluding malignant mesothelioma (MM) [13].

**FIGURE 5:**
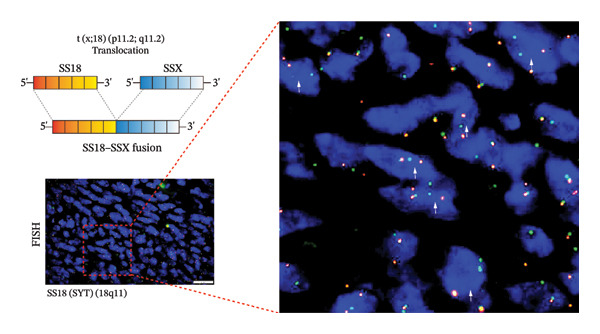
Molecular variants of primary cardiac synovial sarcoma. FISH analysis revealed a chromosomal breakage at the *SS18* (*SYT*) gene locus in the majority of tumor cells. The split signal pattern (white arrows) indicates separation of the red (5′‐SYT) and green (3′‐SYT) probes, confirming a chromosomal translocation involving *SS18* (18q11).

Moreover, FISH analysis demonstrated *SS18–SSX* separation signals in five cases, indicative of the t (X; 18) (p11.2; q11.2) translocation, which generates the *SS18–SSX* fusion oncogene. The split signal pattern, characterized by the separation of the red (5′‐SYT) and green (3′‐SYT) probes (white arrows), was observed (Figure 5). Case 2 yielded a false‐negative FISH result but was subsequently confirmed to harbor the *SS18–SSX1* gene fusion through NGS (Table 1). In addition, our cases underwent surgical intervention (tumor excision) followed by adjuvant chemotherapy with doxorubicin and ifosfamide. Follow‐up data were available for all six novel cases: Four patients remained alive and disease‐free at the time of analysis, whereas two patients had died.

### 3.2. Literature Comparison

#### 3.2.1. Clinical Presentation

Analysis of the entire PCSS dataset demonstrated a significant male predominance (*n* = 83/112, 74.1%), with a male‐to‐female ratio of approximately 3:1; the age distribution (*n* = 112) was skewed, with a mean age of 36.8 years (range: 11–85, interquartile range [IQR]: 25–48). Previous medical and family history was nonspecific, with no identifiable risk factors or hereditary patterns associated with the disease phenotype. Clinical presentation was primarily characterized by cardiopulmonary symptoms, notably dyspnea (*n* = 61, 58.1%), chest pain (*n* = 22, 21.0%), and cough (*n* = 17, 16.2%), with less frequent manifestations including fatigue (*n* = 17, 16.2%), fever (*n* = 11, 10.5%), heart palpitations (*n* = 9, 8.6%), and syncope (*n* = 7, 6.7%) (Table 2). Physical examination findings often included lower extremity edema, jugular venous distention, and pericardial effusion, potentially progressing to cardiac tamponade. Anatomically, the pericardium was the most common site of origin (*n* = 52, 46.4%), followed by the right atrium (*n* = 16, 14.3%), right ventricle (*n* = 14, 12.5%), left ventricle (*n* = 9, 8.0%), and left atrium (*n* = 8, 7.1%), with rarer occurrences in the fibrous skeleton (*n* = 3 [14–16]), atrioventricular (AV) node (*n* = 1 [17]), and interatrial septum (*n* = 1 [18]). Besides, there were eight cases with multiple site involvement. A tumor arising in the AV node region was associated with potential cardiac arrest [17], whereas other locations typically presented with obstructive symptoms.

**TABLE 2:** Clinical symptoms of primary cardiac synovial sarcoma.

Symptoms	Sum^∗^ (percentage^∗∗^)
Cardiorespiratory symptoms	
Short of breath (dyspnea)	61 (58.1%)
Chest pain	22 (21.0%)
Cough	17 (16.2%)
Heart palpitations	9 (8.6%)
Chest tightness	7 (6.7%)
Heart failure	3 (2.9%)
Back pain	2 (1.9%)
Orthopnea	1 (1.0%)
Hemoptysis	1 (1.0%)
Angina	1 (1.0%)
Neurological symptoms	
Syncope	7 (6.7%)
Headache	4 (3.8%)
Dizziness	2 (1.9%)
Hemiplegia	1 (1.0%)
Seizure and loss of consciousness	1 (1.0%)
Convulsion	1 (1.0%)
Lightheadedness	1 (1.0%)
Vision problems	1 (1.0%)
Cardiogenic shock	1 (1.0%)
Gastrointestinal symptoms	
Abdominal distention	4 (3.8%)
Vomit	3 (2.9%)
Nausea	3 (2.9%)
Abdominal pain	3 (2.9%)
Anorexia	2 (1.9%)
Nonspecific systemic symptoms	
Fatigue (weakness)	17 (16.2%)
Fever	11 (10.5%)
Weight loss	5 (4.8%)
Malaise	2 (1.9%)
Others	
Night sweats	4 (3.8%)
Flu‐like symptoms	1 (1.0%)
Hypochondrial discomfort	1 (1.0%)
Hematuria	1 (1.0%)

^∗^No data for seven cases.

^∗∗^ *N* = 105.

#### 3.2.2. Gross and Histopathological Characteristics

**FIGURE 6:**
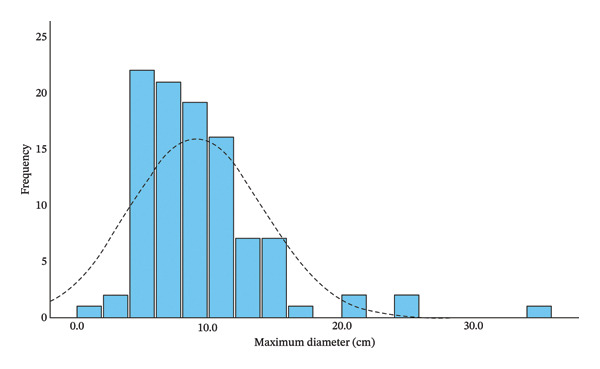
Maximum tumor diameter distribution. The diameter distribution of the tumor indicating a non‐normal distribution with rightward skew (skewness = 2.262; *p* < 0.001), ranged from 1.8 cm to 35.5 cm.

Of the 112 cases in the dataset, valid tumor size data were available for 101 cases. The maximum tumor diameter ranged from 1.8 cm to 35.5 cm, with a mean of 9.13 cm (standard deviation [SD] = 5.05; 95% confidence interval [95% CI]: 8.13–10.12). The tumor size distribution was right‐skewed, as confirmed by the Kolmogorov–Smirnov test (*p* < 0.001) and a skewness coefficient of 2.262 (Figure 6). PCSS cases were categorized into three distinct histopathologic subtypes: monophasic, biphasic, and undifferentiated. Among the 112 cases, definitive histopathological subtyping was available for 92 (82.1%), including the monophasic (*n* = 52, 56.5%), biphasic (*n* = 37, 40.2%), and undifferentiated (*n* = 3, 3.3%). We also performed the statistical analysis to evaluate the associations between histopathological subtypes and clinical variables. Of 112 patients initially included, 20 lacked subtype data and were excluded. Meanwhile, three undifferentiated subtype data were excluded because of insufficient statistical power in the small sample size group. The final analysis is based on 89 complete cases. The *t*‐test demonstrated a significant association between the histopathological subtype of PCSS and age, with biphasic PCSS being more common in older adults (*t* = −2.48, *p* = 0.015). However, the *χ* ^2^ test showed no significant association between subtype and gender (*χ* ^2^ = 0.223; *p* = 0.637) or tumor location (Fisher–Freeman–Halton exact test = 0.997; *p* = 0.825). The Mann–Whitney test demonstrated a significant association between subtype and maximum tumor size, with monophasic PCSS showing a larger tumor diameter (*Z* = −2.191; *p* = 0.028).

#### 3.2.3. Immunohistochemical and Molecular Features

Immunohistochemical analysis was performed in 94 cases with available specimens (Table 3). The results demonstrated the following positivity rates: vimentin (*n* = 49/50, 98%), BCL‐2 (*n* = 48/49, 98%), EMA (*n* = 60/68, 88.2%), CK (*n* = 63/72, 87.5%), and CD99 (*n* = 35/45, 77.8%). Notably, the expression of TLE1 (*n* = 21/21, 100%) and CD56 (*n* = 11/11, 100%) was consistent across all cases, highlighting their diagnostic specificity for PCSS. The Ki‐67 ranged from 5% to 70%, with a mean value of 32.3%. The *χ* ^2^ analysis revealed no significant association between histopathological subtypes and the expression of CK (*p* = 0.128), vimentin (*p* = 0.409), BCL‐2 (*p* = 0.326), EMA (*p* = 0.087), or CD99 (*p* = 0.605). Molecular genetic analysis was performed on cases with available genetic detection data (*n* = 45). The detection methodologies included FISH (*n* = 26, 57.8%), immunofluorescence (IF) (*n* = 3, 6.7%), RT‐PCR (*n* = 13, 28.9%), IHC (*n* = 10, 22.2%), and NGS (*n* = 7, 15.6%). Some studies employed two or more detection methods. Most patients carried the *SS18–SSX* fusion gene transcript, demonstrating the *SS18–SSX* fusion gene as a characteristic feature of PCSS.

**TABLE 3:** Main immunohistochemical marker analysis of primary cardiac synovial sarcoma.

IHC markers	Positive percent (+/sum)
Epithelial differentiation	
Cytokeratin	87.5% (63/72)
P63	0% (0/2)
EMA/BerEP4	88.2% (60/68)
CEA	0.0% (0/3)
Calretinin	42.3% (11/26)
HBME‐1(mesothelin)	0% (0/5)
Mesenchymal differentiation	
Pan‐mesenchymal	
Vimentin	98% (49/50)
Muscular	
SMA	5.4% (2/37)
Desmin	7.3% (3/41)
Calponin	46.2% (6/13)
Myogenin	0% (0/16)
Endothelial	
CD31	11.1% (2/18)
CD34	6.5% (3/46)
WT‐1	20% (3/15)
FLI‐1	75% (3/4)
D2‐40	14.3% (1/7)
ERG	50% (1/2)
Neuroectodermal	
S100	5.9% (3/51)
Melan‐A	0% (0/2)
HMB‐45	0% (0/8)
SOX‐10	0% (0/9)
SOX‐2	100% (5/5)
SYN	40% (2/5)
Hematopoietic	
CD45	0% (0/1)
CD3	0% (0/1)
CD117	20% (1/5)
CD56	100% (11/11)
CD30	100% (1/1)
CD10	0% (0/1)
CD68	0% (0/4)
Others	
CD99	77.8% (35/45)
TLE1	100% (21/21)
STAT6	0% (0/13)
BCL‐2	98.0% (48/49)

#### 3.2.4. Therapeutic Interventions and Prognosis

Surgical intervention, specifically tumor excision, was performed in 89 cases, whereas two cases underwent heart transplantation. Adjuvant chemotherapy was administered to 62 patients. Doxorubicin (*n* = 38, 61.29%) and ifosfamide (*n* = 40, 64.52%) were the most frequently utilized chemotherapeutic agents. Radiotherapy was utilized in 23 cases, primarily for palliative or adjuvant treatments. Notably, five patients received targeted therapies, including immune checkpoint inhibitors (toripalimab and nivolumab) and tyrosine kinase inhibitors (anlotinib, pazopanib, and gefitinib).

Of the total dataset, 90 cases had available prognostic data as follows: no evidence of disease (NED) (*n* = 27, 30%), alive with disease (AWD) (*n* = 23, 25.6%), and died of disease (DOD) (*n* = 40, 44.4%). Local recurrence at diagnosis or postsurgery occurred in 13 PCSS cases (11.6%). Recurrence sites comprised cardiac (*n* = 2) and pulmonary (*n* = 1) involvement. The recurrence interval ranged from 6 months to 7 years (mean 32 months), though this analysis was constrained by heterogeneous follow‐up durations across studies. Survival time data were available for 87 cases, with a median survival time of 26 months (SD = 3.45; 95% CI: 19.23–32.77). The one‐year, two‐year, and five‐year overall survival (OS) rates were 40.23%, 16.09%, and 3.45%, respectively.

## 4. Discussion

PCSS represents an exceptionally rare and aggressive malignant tumor, with limited documentation in the literature [19, 20]. This study presented six newly diagnosed PCSS cases and conducted a literature comparison of 112 previously reported cases, representing the largest analysis to date, thereby further elucidating the clinicopathological and molecular characteristics of PCSS. Demographically, the peak incidence of PCSS, consistent with that of synovial sarcoma [21] in general, was observed in the third to fourth decades of life. PCSS demonstrated a male predilection, with a male‐to‐female ratio of approximately 3:1. Anatomically, the pericardium was the most common site of involvement, observed in 46.4% of cases. Tumors frequently presented as large masses, with a mean maximum diameter of 9.13 cm. Clinically, PCSS frequently presents with nonspecific cardiopulmonary symptoms, including dyspnea, chest pain, and cough [22]. These manifestations are primarily attributable to chronic pericardial effusion caused by PCSS [23], particularly in cases with pericardial involvement. Fever, reported in 10.5% of PCSS cases, may represent a nonspecific systemic manifestation potentially attributable to pathogenic infections or neoplastic fever. Cancer cells can release pyrogenic cytokines and small proteins, inducing fever by directly acting on the anterior preoptic nuclei of the hypothalamus [24]. Osaka et al. [25] reported a case of synovial sarcoma in which interleukin‐1α (IL‐1α) expression was identified as a causative factor for fever. Complete tumor resection resulted in the resolution of fever, supporting the role of IL‐1α in neoplastic fever. Furthermore, in certain cases, patients may develop rare complications, including hematuria secondary to thrombocytopenia [26], Budd–Chiari syndrome [27], and unstable angina [28] resulting from mechanical compression by cardiac tumors, as well as thromboembolic events such as cardioembolic stroke [18] or seizure [29]. Besides, PCSS may manifest as an isolated pericardial effusion in the absence of discernible pericardial masses [30], posing significant diagnostic challenges.

A systematic meta‐analysis [31] incorporating 24 studies demonstrated a significant association between several risk factors and the development of soft tissue sarcomas (STSs). Among the factors examined, smoking (*p* = 0.23), genetic predisposition (*p* = 0.13), chronic inflammation (*p* = 0.20), and toxins (*p* = 0.14) were identified, though none met statistical significance. Likewise, well‐established risk factors for PCSS, a rare STS subtype, remain undefined. Evidence suggests a strong genetic component, particularly its association with the chromosomal translocation t (X; 18) (*SS18–SSX*). However, it remains unknown whether this mutation occurs sporadically or follows a specific chain of events [32]. The resulting *SS18::SSX* fusion gene drives synovial sarcoma oncogenesis [33]. Hill et al. [34] demonstrated *SS18::SSX*‐mediated synovial sarcoma transformation using a mesenchymal stromal cell (MSC)–specific CreERT2 line expressing *SS18::SSX.* In their mouse model, *SS18::SSX* expression leads to 100% penetrant SS development. Their work further revealed that synovial sarcoma arises through the stepwise loss of mature fibroblastic features and the reactivation of an embryonic mesenchymal program. These findings suggest that synovial sarcoma may originate from a fibroblastic population.

Histologically, the monophasic subtype, which was more prevalent, was characterized by fascicular arrangements of spindle cells, whereas the biphasic subtype was characterized by alternating epithelial and spindle cell components. The biphasic PCSS was more common in older adults. Notably, consistent with the findings of Chen et al. [35], regarding synovial sarcoma histopathological subtypes, our analysis revealed no significant association between the histopathological subtype of PCSS and gender or tumor location. Immunophenotypically, PCSS exhibited high TLE1/CD56 specificity and vimentin/BCL‐2 sensitivity, collectively aiding diagnosis. Given the potential for false‐negative FISH results, we recommend proceeding with NGS testing to enhance diagnostic accuracy in cases where FISH is negative, but clinical suspicion remains high and tumor expression of TLE1 and CD99 is present. Conversely, NGS testing is unnecessary when FISH yields a positive result. Based on anatomical predilection sites and histopathological features, the differential diagnosis of PCSS encompasses angiosarcoma, fibrosarcoma, leiomyosarcoma, malignant solitary fibrous tumor (MSFT), rhabdomyosarcoma (RMS), undifferentiated pleomorphic sarcoma (UPS), malignant peripheral nerve sheath tumor (MPNST), and MM. Key distinguishing features are summarized in Table 4 [36–45].

**TABLE 4:** Differential diagnoses based on immunohistochemistry and molecular characteristics.

Diagnosis	Immunohistochemistry	Molecular characteristics
SS	CK+, TLE1+, EMA+, vimentin+, BCL‐2, S100−, CD34−	*SS18–SSX* fusion
Angiosarcoma	CD31+, ERG+, CD34+, vWF+	No specific fusion
Fibrosarcoma	CK−, EMA−, S100−, desmin−, SMA−, CD34−, STAT6−	*COL1A1–PDGFB* fusion possible
Leiomyosarcoma	SMA+, desmin+, caldesmon+, CK−	No specific fusion
MSFT	STAT6+, CD34+, BCL‐2+, CK−	*NAB2–STAT6* fusion possible
RMS	MyoD1+, myogenin+, desmin+, CK−	*PAX3/7–FOXO1* fusion‐positive (alveolar type) and fusion‐negative (embryonal type)
UPS	S100−, desmin−, SMA−, CK−, EMA−, CD34−, STAT6−	No specific fusion
MPNST	S100+, SOX10+, H3K27me3 loss, CK−	*NF1* mutations possible
MM	Calretinin+, WT‐1+, CK5/6+, EMA+, D2‐40+, BerEP4−, MOC‐31−, CEA−, TTF1−	*BAP1* deletion possible, *CDKN2A (p16)* homozygous deletion possible

Abbreviations: MM = malignant mesothelioma, MPNST = malignant peripheral nerve sheath tumor, MSFT = malignant solitary fibrous tumor, RMS = rhabdomyosarcoma, SS = synovial sarcoma, UPS = undifferentiated pleomorphic sarcoma.

In the literature comparison, 89 patients underwent surgery, 62 received chemotherapy, and 23 underwent local radiotherapy. Given the rarity of PCSS, standardized treatment protocols remain undefined, and current clinical practice largely follows guidelines for STSs [46]. For patients with localized and resectable tumors, complete surgical resection (R0) is the cornerstone of first‐line therapy, as it offers the best potential for cure and long‐term survival. Our analysis found a postoperative recurrence rate of 11.6%, which is relatively low for a malignant tumor and supports the efficacy of complete resection. This is further corroborated by a recent case reported by Nagasawa et al. [47], in which a patient with a tumor invading the right atrial free wall achieved complete resection and showed no recurrence at 6‐month follow‐up without adjuvant therapy. These findings collectively underscore that achieving a complete resection is a critical favorable prognostic factor. The first‐line regimen typically comprises surgical resection followed by risk‐adapted combination chemotherapy with doxorubicin (75 mg/m^2^ via 72‐h continuous intravenous infusion or in divided doses over 3 consecutive days) and ifosfamide (2.5 g/m^2^/day for 5 days), administered every 21 days for 2–3 cycles. The decision for adjuvant chemotherapy should be individualized based on postoperative pathological risk assessment. For disease progression after first‐line therapy [48], gemcitabine plus docetaxel is recommended. In *SS18–SSX*‐negative cases, comprehensive molecular profiling via NGS should be pursued to identify actionable alterations (e.g., NTRK fusions), warranting trial of repotrectinib.

Despite aggressive approaches, the prognosis remained guarded. Van der Mieren et al. [49] documented the longest recorded survival (14 years) in PCSS patients who underwent aggressive multimodal therapy and rigorous surveillance. Nevertheless, the median survival time for patients with PCSS in this study was 26 months.

However, some limitations must be considered. The inherent rarity of PCSS limits sample size despite our large aggregate dataset. Case‐based data collection introduces potential bias and incomplete datasets (e.g., variable treatment details and missing follow‐up). What is more, the lack of standardized treatment protocols resulted in diverse therapeutic approaches, making robust comparisons of specific regimens difficult.

In the future, research should focus on elucidating the specific oncogenic mechanisms driven by *SS18–SSX* variants within the cardiac microenvironment, as well as identifying potential unique cooperating mutations in PCSS. Collaborative initiatives to establish prospective registries are essential to address sample size limitations and to standardize data collection regarding clinical presentation, diagnostic approaches, treatment modalities, and patient outcomes. The development of preclinical models and the conduct of clinical trials assessing targeted therapies and immunotherapeutic strategies in molecularly defined PCSS subsets are critical for enhancing patient outcomes.

## 5. Conclusion

PCSS represents a rare and aggressive subset of synovial sarcoma, predominantly affecting young to middle‐aged males and presenting with nonspecific cardiopulmonary symptoms. Given its poor prognosis, early histopathological and molecular characterization, coupled with personalized therapeutic strategies, is essential for improving outcomes. In conclusion, although our study provides insights into PCSS, significant challenges remain. Multidisciplinary collaboration among cardiology, oncology, and molecular pathology is imperative to advance our understanding of PCSS and transform this lethal disease into a manageable condition.

## Author Contributions

Yi‐Xiang Cai: conceptualization, data curation, formal analysis, investigation, methodology, visualization, writing–original draft, and writing–review and editing. Sheng‐Jun Liu: conceptualization, data curation, investigation, methodology, visualization, writing–original draft, and writing–review and editing. Qi Sun: conceptualization, data curation, formal analysis, investigation, and methodology. Hui Li: conceptualization, funding acquisition, project administration, resources, supervision, and validation. Xiao‐Le Meng: conceptualization, funding acquisition, methodology, project administration, resources, supervision, validation, and writing–review and editing.

## Funding

This study was supported by the National Natural Science Foundation of China–Young Scientists Fund (No. 82503101); Fujian Provincial Health Science and Technology Program–Young and Middle‐Aged Backbone Talent Training Project (No. 2025GGA104); Xiamen Health and Wellness High‐Quality Development Science and Technology PlanYoung and Middle‐Aged Key Talents Training Program (No. 2024GZL‐GG05); Natural Science Foundation of Xiamen, China (No. 3502Z20227123); and Young Investigator Research Program of Xiang’an Hospital of Xiamen University (No. XM02030005).

## Ethics Statement

This study was based on present cases and review of published literature and complies with the ethical standards of the Helsinki Declaration and was approved by the appropriate ethical committees. Written informed consent was obtained from the patient for publication of this case report and accompanying images. A copy of the written consent is available for review by the editor in chief of this journal on request.

## Conflicts of Interest

The authors declare no conflicts of interest.

## Supporting Information

Supporting figure 1 Graphical abstract of primary cardiac synovial sarcoma. Supporting table 1. Cases included in the literature comparison.

## Supporting information


**Supporting Information** Additional supporting information can be found online in the Supporting Information section.

## Data Availability

The datasets generated and analyzed during the present study are available from the corresponding authors on reasonable request.
