# Supplementary material for: Primary Cardiac Synovial Sarcoma (PCSS): Clinicopathologic Features of 6 Cases and Literature Comparison
Source: Cardiol Res Pract. 2026 Apr 13;2026:5272035. doi: 10.1155/crp/5272035 (PMC13074430; doi:10.1155/crp/5272035)
Supplement: Supplementary file 1 — Supporting Information Additional supporting information can be found online in the Supporting Information section. [file CRP-2026-5272035-s001.zip › Supplementary figure 1.pdf]

Graphical Abstract

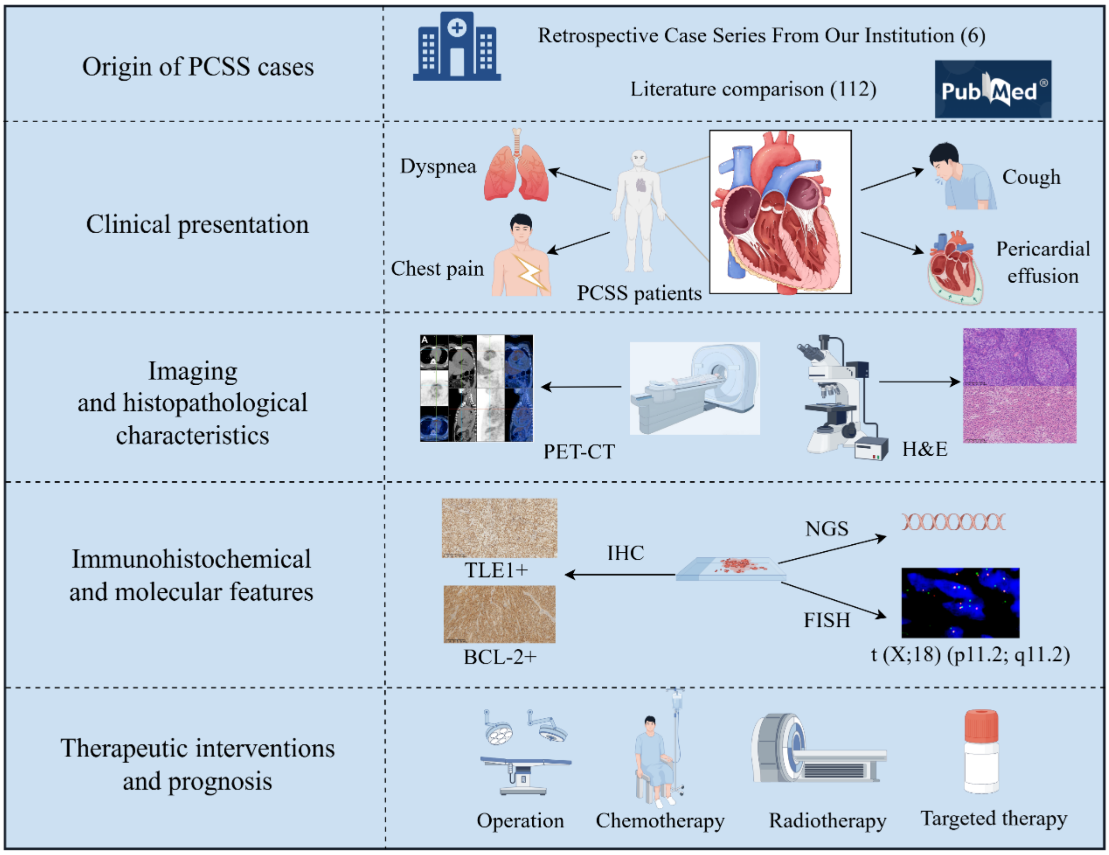

Supplementary fig.1 Graphical abstract of primary cardiac synovial sarcoma.
