# Supplementary material for: Primary Cardiac Synovial Sarcoma (PCSS): Clinicopathologic Features of 6 Cases and Literature Comparison
Source: Cardiol Res Pract. 2026 Apr 13;2026:5272035. doi: 10.1155/crp/5272035 (PMC13074430; doi:10.1155/crp/5272035)
Supplement: Supplementary file 1 — Supporting Information Additional supporting information can be found online in the Supporting Information section. [file CRP-2026-5272035-s001.zip › Supplementary table 1.docx]

**Supplementary table 1. Cases included in the literature comparison**

| Patient number | Publication year | Authors | Prognosis | Survival time |
| --- | --- | --- | --- | --- |
| 1 | 1988 | E A Sheffield et al. | DOD* | 6 months |
| 2 | 1990 | R Siebenmann et al. | DOD | 3 months |
| 3 | 1992 | A P Burke et al. | NA | NA |
| 4 | 1994 | C M Karn et al. | DOD | 1 months |
| 5 | 1996 | L L Constantinou et al. | DOD | 5.5 months |
| 6 | 1997 | A G Nicholson et al. | NED | >10 months |
| 7 | 1998 | M Fujioka et al. | DOD | 8 months |
| 8 | 1998 | K Langner et al. | NA | NA |
| 9 | 1999 | A V Donsbeck et al. | NA | NA |
| 10 | 1999 | S H Bean et al. | AWD | >14 months |
| 11 | 1999 | S Oizumi et al. | DOD | 7 months |
| 12 | 1999 | N Al-Rajhi et al. | NED | >12 months |
| 13 | 2000 | B Bittira et al. | DOD | NA |
| 14 | 2003 | A K AnaNA et al. | AWD | >13 months |
| 15 | 2003 | Teresa T McGilbray et al. | NA | NA |
| 16 | 2004 | Hans Martin Hazelbag et al. | DOD | 1 months |
| 17 | 2004 | Triantafyllia Koletsa et al. | AWD | >4 months |
| 18 | 2004 | Gerry Van der Mieren et al. | DOD | 14 years |
| 19 | 2004 | Masaaki Yano et al. | NED | >12 months |
| 20 | 2005 | Dylan V Miller et al. | NED | >6 months |
| 21 | 2005 | Hugues Bégueret et al. | NA | NA |
| 22 | 2006 | Sylvio Carvalho Provenzano et al. | NED | >18 months |
| 23 | 2006 | Maria Luisa C Policarpio-Nicolas et al. | NA | NA |
| 24 | 2007 | Qiong Zhao et al. | AWD | >3 months |
| 25 | 2007 | SaNAra N Hing et al. | DOD | 31 months |
| 26 | 2007 | Brian Boulmay et al. | AWD | >4 months |
| 27 | 2008 | Paul J Zhang et al. | NED | >13 months |
| 28 | 2008 | Paul J Zhang et al. | NA | NA |
| 29 | 2009 | Narain Moorjani et al. | AWD | >2 months |
| 30 | 2009 | A Korula et al. | NA | NA |
| 31 | 2009 | H Katakura et al. | DOD | 79 days |
| 32 | 2010 | Xiayi Lv et al. | AWD | >22 months |
| 33 | 2010 | Muhammad Talukder et al. | DOD | 1 months |
| 34 | 2011 | Rangreze Imran et al. | DOD | 26 months |
| 35 | 2011 | F Akerström et al. | DOD | 6 months |
| 36 | 2011 | Yuki Yokouchi et al. | NED | >9 months |
| 37 | 2012 | Li-yang Tao et al. | AWD | >12 months |
| 38 | 2012 | Yufan Cheng et al. | DOD | 27 months |
| 39 | 2012 | Yufan Cheng et al. | AWD | >2 months |
| 40 | 2012 | Yufan Cheng et al. | NA | NA |
| 41 | 2012 | SarathchaNAra Kodikara et al. | NA | NA |
| 42 | 2013 | Xia Wu et al. | AWD | >21 months |
| 43 | 2013 | Tarik Chekrine et al. | NED | >32 months |
| 44 | 2013 | Lijuan Yin et al. | NED | >3 months |
| 45 | 2014 | Habib Khan et al. | NA | NA |
| 46 | 2014 | Michishige Ohzeki et al. | NED | >5 months |
| 47 | 2014 | Guang-Won Seo et al. | AWD | NA |
| 48 | 2014 | Michael Wolf et al. | AWD | 3 months |
| 49 | 2014 | Mitsuru Yoshino et al. | AWD | >1 year |
| 50 | 2014 | Prajakta Phatak et al. | AWD | >18 months |
| 51 | 2015 | Prasad Eswaran et al. | NA | NA |
| 52 | 2015 | Imran Khan et al. | NED | >24 months |
| 53 | 2015 | Evrim Ozmen et al. | NED | >11 months |
| 54 | 2015 | Anil Sharma et al. | NA | NA |
| 55 | 2015 | Zhen Huo et al. | NED | >18 months |
| 56 | 2015 | Joshua Goldblatt et al. | AWD | >4 months |
| 57 | 2015 | Edvin Prifti et al. | AWD | >1 months |
| 58 | 2016 | Lauren R Schaffer et al. | NA | NA |
| 59 | 2016 | Hyo Chul Youn et al. | DOD | 1 week |
| 60 | 2017 | Mahmood Hosseinzadeh Maleki et al. | NED | >15 months |
| 61 | 2017 | Berta Vega HernáNAez et al. | AWD | > 7 months |
| 62 | 2017 | Avani S Jain et al. | NA | NA |
| 63 | 2017 | Kelechukwu U Okoro et al. | DOD | 1 day |
| 64 | 2018 | Antonella Coli et al. | DOD | 18 months |
| 65 | 2018 | Antonella Coli et al. | DOD | 32 months |
| 66 | 2018 | Wael Braham et al. | NED | >8 years |
| 67 | 2018 | Nicolas De Hous et al. | NED | >12 months |
| 68 | 2018 | Shusaku Maeda et al. | DOD | 36 months |
| 69 | 2018 | Hiroaki Osada et al. | NED | >13 months |
| 70 | 2019 | Jose Duran-Moreno et al. | DOD | 6 months |
| 71 | 2019 | Guodong Zhang et al. | NED | >36 months |
| 72 | 2020 | Jessica Napuri et al. | DOD | 1 months |
| 73 | 2020 | Fei Teng et al. | DOD | 12 months |
| 74 | 2020 | Fei Teng et al. | AWD | 16 months |
| 75 | 2020 | Fei Teng et al. | NED | 12 months |
| 76 | 2020 | Fei Teng et al. | DOD | 3 months |
| 77 | 2020 | Fei Teng et al. | DOD | 13 months |
| 78 | 2020 | Kirsten Y Wong et al. | NED | >15 months |
| 79 | 2020 | Akbarshakh Akhmerov et al. | NED | >6 months |
| 80 | 2020 | Chang Liu et al. | NA | NA |
| 81 | 2021 | Dhiren Shah et al. | NED | >3 months |
| 82 | 2021 | Safia Ouarrak et al. | DOD | 8 months |
| 83 | 2021 | Felipe Matsunaga et al. | NA | NA |
| 84 | 2021 | Ammar Farook Chapra et al. | DOD | 3 days |
| 85 | 2021 | Mirosława Püsküllüoglu et al. | DOD | 26 months |
| 86 | 2021 | Shahryar G Saba et al. | NA | NA |
| 87 | 2021 | Sneha Thatipelli et al. | DOD | NA |
| 88 | 2022 | Toktam Alirezaei et al. | DOD | 15 months |
| 89 | 2022 | Efstathios D Pagourelias et al. | NA | NA |
| 90 | 2022 | Adam J Eqbal et al. | DOD | 30months |
| 91 | 2022 | Ingeborg M Keeling et al. | NED | >8months |
| 92 | 2022 | Danielle M Mullis et al. | NED | >9momths |
| 93 | 2022 | Klaudiusz Stoklosa et al. | NED | >1 year |
| 94 | 2022 | Alice L Zhou et al. | NED | >4months |
| 95 | 2023 | Tomás F Cianciulli et al. | DOD | 7years |
| 96 | 2023 | Sarvesh Kumar et al. | NA | NA |
| 97 | 2023 | Fang He et al. | NA | NA |
| 98 | 2023 | Hira Irfan et al. | DOD | 17 months |
| 99 | 2023 | Hongrui Jin et al. | NA | NA |
| 100 | 2023 | Busra Yaprak Bayrak et al. | DOD | 21months |
| 101 | 2024 | Ramanish Ravishankar et al. | AWD | >30 months |
| 102 | 2024 | Xing Zhang et al. | DOD | 8 months |
| 103 | 2024 | Mixia Li et al. | DOD | 6 months |
| 104 | 2024 | Nie Xu et al. | AWD | >15 months |
| 105 | 2024 | Abhijit RaviNAran et al. | AWD | >1 year |
| 106 | 2024 | Binyue Wang et al. | DOD | 26 months |
| 107 | 2024 | X H Liu et al. | NA | NA |
| 108 | 2024 | X H Liu et al. | DOD | 7 months |
| 109 | 2024 | X H Liu et al. | DOD | 3 months |
| 110 | 2024 | X H Liu et al. | DOD | 13 months |
| 111 | 2024 | X H Liu et al. | NED | >1 year |
| 112 | 2024 | Tomonori Kawasaki et al. | AWD | >14 months |
| *AWD, alive with disease; DOD, died of disease; NED, no evidence of disease; NA, not applicable. | | | | |
